# 

**Published:** 1920-05-08

**Authors:** 


### THE FUNERAL SERVICE.

Viscount Knollys represented Her Majesty Queen Alexandra at the Choral Service at the Parish Church, Holy Trinity, Bishop's Road, Paddington, which was jointly conducted by the Rev. Charles Knight, the vicar, and the Rev. Halford Lawson (nephew). The hymns chosen were, "Lead, kindly Light," "Lead us, Heavenly Father, lead us," and "Now the labourer's task is o'er." The rendering of Ps. xxiii, "The Lord is my Shepherd," seemed to crown the service, and to be completely in keeping with the spirit which imbued it.

The principal mourners were :—Father Burdett, S.J., and Mr. Osbert Burdett (sons), Mrs. M. L. Gwyer (daughter), Miss Mary Burdett (sister), Mrs. Halford Burdett and Masters Chichele, Derek, and Gay Burdett (widow and sons of the eldest son, Captain Halford Burdett), Mr. Maurice L. and Miss Miranda Gwyer (son-in-law and granddaughter), Mrs. Osbert Burdett (daughter-in-law), Mr. E. P. and Miss Shute (brother-in-law and sister-in-law), the Rev. Halford Lawson and Mr. H. P. Lawson (nephews).

Viscount Knollys (Lord in Waiting) represented Her Majesty Queen Alexandra.

Amongst those present were : Lord Somerleyton (King Edward's Hospital Fund); the Hon. Sir Arthur Stanley (the College of Nursing and British Red Cross Society); Mr. Robert Holland Martin (Vice-President, Hospital Sunday Fund); Mr. J. W. Facey (assistant secretary, in the absence of Mr. Dick, secretary of the Royal National Pension Fund for Nurses); Miss Rosa Smith (secretary, Junius S. Morgan Benevolent Fund); Sisters G. Bremner, Crowther, McCammon, E. H. Paterson, and L. Wood (Nurses' Co-operation); Sir James Harrison (the League of Mercy); Mr. Arnold James (Hospital Sunday Fund); Mr. R. A. Owthwaite and Mr. J. Courtney Buchanan (British Hospitals Association) : Miss A. Eva Milnes (secretary, League of Mercy); Miss Sheriff MacGregor (College of Nursing); Sir Perceval Nairne; Sir George and Lady Makins; Sir William Plender; Sir Charles Cook; Miss Rosalind Paget (Midwives Institute); Mr. P. Michelli (Seamen's Hospital): Mr. G. Q. Roberts (St. Thomas's Hospital); Mr. F. Morgan Bryant; Miss Hogg and Miss MacManus (matron and assistant-matron, Guy's Hospital); Miss Lloyd Still (matron, St. Thomas's Hospital); Miss Cox-Davies (Royal Free Hospital); Miss Verrall (Headquarters, V.A.D.); Mr. Richard Kershaw (Central London Throat, Nose and Ear Hospital); Mr. Woolley (Royal Waterloo Hospital); Mr. Leonard Cohen; Mr. J. East (London County Westminster and Parr's Bank and the Miller General Hospital); Mr. J. S. Spong (representing the Governors of St. Bartholomew's Hospital); Mr. S. M. Quennell (Westminster Hospital); Mr. H. Wigg (Hampstead General Hospital); Dr. F. W. Cock; Mr. Penfold; Miss J. Elliot; Mrs. Chalmers Mitchell (representing Dr. Chalmers Mitchell); Mr. Henry Gascoigne Maurice (Ministry of Agriculture); Mr. J. Cameron-Head; Mrs. Arnold Lawson; Mrs. Alcyn Lawson; Mr. Claude Pemberton Leach; Col. and Mrs. Huntly; Mrs. Pritchard Binnie; Mr. W. E. Wallace; Mr. George D'Oyley Hutchins; Miss Gertrude Leigh; Dr. E. L. Ash; Mr. and Mrs. C. J. Bill; Mr. and Mrs. Clifford Crewe; Miss L. Clara Fisher (the first nurse to join the Royal National Pension Fund); Miss J. Skillman ("Sister Hope" of St. Bartholomew's Hospital); Sister Craig; Miss Christie; Mr. Lawrence Tanner; Mr. Geoffrey Lawson; Mr. and Mrs. Arthur Brown; Mrs. Drew; Miss Slaughter; Mrs. Awdry; Miss Chaplin; Mrs. Gibson (representing Dr. Gibson); Miss Croke Robinson; Mr. H. E. Wortham; Miss E. M. Litten (Sir Henry's private secretary for many years); Dr. F. A. Knott (The Hospital); Mrs. Wilcox and Miss B. Wilcox (*The Nursing Mirror*); Mr. H. E. Smithers, Mr. A. D. Rand, Mr. G. B. Garnham, and Mr. C. Coram (General Manager and members of the staff of The Scientific Press), and Miss E. L. Shepherd.

### Passing Events and Topics.

### Ambulance Train for Poultry Demonstration.

The Great Eastern Railway are stated to be adapting a number of ambulance carriages to be used shortly for the purpose of poultry demonstration. This "Poultry Train," when fitted up, is proposed to be run to various centres in East Anglia, where lectures, etc., will be given by experts upon the keeping of poultry, goats, bees, rabbits, etc.

### Public Pharmacists' Association.

The Public Pharmacists' Association held a meeting on March 28, 1920, at St. Bride Institute, London, when Mr. J. L. P. Hollingworth, F.L.S., M.P.S., presided, and delivered a lecture on "India and Its Customs." A representative gathering of pharmacists engaged at voluntary hospitals and public institutions attended the meeting.

### Matrons' and Sisters' Appointments.

SPRINGFIELD HOSPITAL, ROCHDALE.—Miss Mary Beard has been appointed ward sister. She was trained at the City of London Infirmary, and has since been staff nurse at Southampton Eye Hospital and sister at the Memorial Hospital, Ludhiana, Punjab, India.

TUBERCULOSIS INSTITUTION, GLOUCESTER.—Miss Ethel M. Jackson has been appointed matron. She was trained at the Royal Devon and Exeter Hospital, and has since been matron of the Hawksmoor Sanatorium, Devon.

HOSPITAL FOR WOMEN SUFFERING FROM VENEREAL DISEASE, SHEFFIELD STREET, KINGSWAY, W.C.—Miss A. N. Timbrell, A.R.R.C., has been appointed matron. She was trained at Guy's Hospital, has been sister at the Government Hospital, Gold Coast, night sister at the Cumberland Infirmary, and matron of the Bangkok Nursing Home, the Lowestoft and North Suffolk Hospital, and matron of the Lowestoft Nursing Association.

ULVERSTON AND DISTRICT COTTAGE HOSPITAL.—Mrs. A. M. Flegun has been appointed nurse-matron. She was trained at the Withington Hospital, West Didsbury, has been sister-in-charge of St. George's Military Hospital, Stockport, and sister-in-charge of the operating theatre at South Manchester Hospital.

NEW END HOSPITAL, HAMPSTEAD.—Miss J. N. Jackson, O.B.E., R.R.C., has been appointed matron. She was trained at St. Bartholomew's Hospital and at the Rotunda Hospital, Dublin, and has been acting matron of the New Hospital for Women, Euston Road, assistant matron of Kennington Infirmary and the Royal Sussex County Hospital, Brighton, and matron of the Royal Surrey County Hospital, Guildford, and of the South African Hospital, Richmond.

CITY OF LONDON HOSPITAL FOR DISEASES OF THE CHEST, VICTORIA PARK, E.—Miss Laura E. Webb has been appointed sister. She was trained at the Bermondsey and Rotherhithe Hospital, has been staff nurse at the Western Fever Hospital, Fulham, sister at the South-Western Fever Hospital, Stockwell, the North-Eastern Fever Hospital, Tottenham, at the Military Hospital, Aldershot, at the No. 3 British General Hospital, Basra, Mesopotamia, and at the No. 19 General Hospital, Alexandria, serving in Queen Alexandra's Imperial Military Nursing Service.

NORTH-EASTERN FEVER HOSPITAL, SOUTH TOTTENHAM.—Miss Ethel Smith, Miss Edith Annie Shaw, and Miss Maude Alice Robinson have been appointed sisters. Miss Smith was trained at Holborn Infirmary, Highgate, and has served with the British Red Cross Society and Queen Alexandra's Imperial Military Nursing Service Reserve. Miss Shaw was trained at the Royal Victoria Hospital, Newcastle-on-Tyne, and at the Northern Convalescent Fever Hospital, Winchmore Hill, and has served in the Territorial Force Nursing Service. She is a member of the College of Nursing. Miss Robinson was trained at the Hastings Borough Hospital and at Bethnal Green Infirmary, and has served as sister with the British Red Cross Society at Manchester and Hull.

### Royal Southern Hospital, Liverpool.

MR. ALLAN NALDRETT has resumed his duties at the Liverpool Royal Southern Hospital after an absence of five years and seven months, during which he has been serving in the Royal Army Medical Corps, latterly at the 1st Western General Hospital, with rank as Captain and Quartermaster.

### Chicken-Pox at Glasgow.

IN view of an outbreak of smallpox, and the danger of certain cases being treated as chicken-pox, Glasgow Corporation, as a precautionary measure, has made the latter a notifiable disease as from May 1.

### The College of Nursing (Limited by Guarantee).

### EAST LANCASHIRE LOCAL CENTRE.

THE second annual meeting of the East Lancashire Centre was held at the Manchester Royal Infirmary on Tuesday, April 27, 1920, at 7 P.M., Miss Sparshott, C.B.E., R.R.C., presiding. About 170 members were present.

The Committee were elected by ballot. Miss Sparshott, C.B.E., R.R.C., was re-elected President, and Mrs. Burgess, M.B.E., local representative. Miss Pilgrim, who had been hon. treasurer during the past year, resigned the post owing to her work necessitating her frequent absence from Manchester, and Miss Earl was elected hon. secretary and treasurer combined.

The relation of the Hours of Employment Bill, at present before Parliament, to the nursing profession was considered, and a lengthy discussion ensued. Arising out of a communication received from the Liverpool Centre, the question of provincial representation on the College Council was discussed, and whilst it was felt that the provinces were not fairly represented, and that it ought not to be necessary for Centres to combine forces in order to get members returned, it was realised that it was too late for any alteration to be made before the impending election.

Miss Sheriff Macgregor gave a very interesting address on the objects of the College, and also a *résumé* of the work it had done. The East Lancashire Centre are proud to be the first Centre to welcome the newly-appointed Organising Secretary of the College of Nursing.

The total membership of the East Lancashire Centre at the present time is 367.

### LIVERPOOL CENTRE.

THE second annual meeting of the Liverpool Centre was held on Wednesday, April 28, at the Royal Infirmary, Miss Cummins, R.R.C., in the chair. The Hon. Secretary, Miss Worsley, presented the report, which stated that the membership had increased considerably during the past year. The branch had followed closely the policy of the College of Nursing, and the members had greatly benefited by the advice and experience of Miss Cummins, who as President and representative on the Board of the Council of the College had been able to keep them in touch with the Council's progress. The Centre had forwarded to the College £50 for the Chair of Nursing.

An interesting address was given by Miss Sheriff-Macgregor, Organising Secretary of the College of Nursing, on the aims and objects of the College of Nursing. This was followed by a discussion on the Hours of Employment Bill, when forcible arguments against the Bill were put forward by Miss Golding on private nursing and Miss Drysdale on district nursing. It was finally unanimously resolved that the members of the Liverpool Centre of the College of Nursing do not agree that the nursing profession should come under the Hours of Employment Bill.

#### THE CLUB.

It was reported that the Club was running satisfactorily, and the number of visits had reached 600. A vote of cordial thanks was passed to the Finance Committee who are responsible for providing the funds for the Club.

### BRIGHTON AND HOVE CENTRE.

THE Annual Members' Meeting was held in the Club at No. 45 Marine Parade, Brighton, on April 21, Miss K. Scott, R.R.C., presiding. The annual report and financial statement were presented and adopted, and an auditor appointed for the coming year. The Executive Committee for the coming year was appointed, all the previous members being re-elected, and two new members, Miss Haines and Miss Stride, were also appointed. The interim report re the Hours of Employment Bill was read and freely discussed.

While agreeing that nurses should have shorter hours and be paid a sufficient salary to live in comfort and have no anxiety about the future, the members were unanimous in their strong disapproval of a compulsory eight-hour day. They were emphatically of the opinion that it is in contradiction to the true spirit of nursing, both in institutions and in private work, and is putting the profession on a very low basis. Resolutions to this effect were carried *nem. con.*, and the Secretary was instructed to forward copies of these to the Secretary of the College. Miss Scott, R.R.C., was re-elected President of the Centre, and Miss A. M. Latham Hon. Secretary and Treasurer.

